# Newly Diagnosed Diabetes in Patients with COVID-19: Different Types and Short-Term Outcomes

**DOI:** 10.3390/tropicalmed6030142

**Published:** 2021-08-02

**Authors:** Alaa A. Farag, Hassan M. Hassanin, Hanan H. Soliman, Ahmad Sallam, Amany M. Sediq, Elsayed S. Abd elbaser, Khaled Elbanna

**Affiliations:** 1 Internal Medicine Department, Faculty of Medicine, Zagazig University, Zagazig 44519, Egypt; drhassan_h99@yahoo.com (H.M.H.); aboamro76@yahoo.com (K.E.); 2 Community Medicine Department, Faculty of Medicine, Suez Canal University, Ismailia 41522, Egypt; hananhasan81@yahoo.com; 3 Clinical Pathology Department, Faculty of Medicine, Zagazig University, Zagazig 44519, Egypt; nancyabdelhamid@yahoo.com (A.S.); amany_mohy2006@yahoo.com (A.M.S.); 4 Tropical Medicine Department, Faculty of Medicine, Zagazig University, Zagazig 44519, Egypt; dr.sayedsaad79@gmail.com

**Keywords:** COVID-19, new-onset DM, severe infection, mortality

## Abstract

A great global concern is currently focused on the coronavirus disease 2019 (COVID-19) pandemic and its associated morbidities. The goal of this study was to determine the frequency of newly diagnosed diabetes mellitus (DM) and its different types among COVID-19 patients, and to check the glycemic control in diabetic cases for three months. After excluding known cases of DM, 570 patients with confirmed COVID-19 were studied. All participants were classified as non-diabetic or newly discovered diabetic. According to hemoglobin A1c (HbA1c) and fasting insulin, newly discovered diabetic patients were further classified into pre-existing DM, new-onset type 1 DM, and new-onset type 2 DM. Glycemic control was monitored for three months in newly diagnosed diabetic patients. DM was diagnosed in 77 patients (13.5%); 12 (2.1%) with pre-existing DM, 7 (1.2%) with new-onset type 1 DM, and 58 (10.2%) with new-onset type 2 DM. Significantly higher rates of severe infection and mortality (*p* < 0.001 and *p =* 0.046) were evident among diabetic patients. Among survived diabetic patients (*n* = 63), hyperglycemia and the need for anti-diabetic treatment persisted in 73% of them for three months. COVID-19 was associated with a new-onset of DM in 11.4% of all participants and expression of pre-existing DM in 2.1% of all participants, both being associated with severe infection. COVID-19 patients with newly diagnosed diabetes had high risk of mortality. New-onset DM persisted for at least three months in more than two-thirds of cases.

## 1. Introduction

In December 2019, in Wuhan (China), the first cases of severe pneumonia of unknown origin were reported [1]. The causative organism has been identified as a new enveloped RNA beta-coronavirus, and it was later named severe acute respiratory syndrome coronavirus 2 (SARS-CoV-2) [2]. Early in 2020, the World Health Organization (WHO) declared coronavirus disease 2019 (COVID-19) as a global pandemic. Globally, as of 7 July 2021, there have been 184,324,026 confirmed cases of COVID-19, including 3,992,680 deaths, reported to WHO [3].

Diabetes is a common chronic metabolic disease, and one of the major causes of morbidity and mortality, which leads to huge health and financial burden worldwide. Patients with diabetes have an increased risk of severe complications, including severe acute respiratory syndrome (SARS) and multi-organ failure [4].

There is a two-way relationship between COVID-19 and DM [5]. In the first way, diabetes is associated with a poor COVID-19 prognosis [6]. In the other way, new-onset DM and severe complications of pre-existing DM, including diabetic ketoacidosis (DKA) and hyperosmolarity, have been reported in patients with COVID-19 [7].

Severe acute respiratory syndrome coronavirus 2 may enter the pancreatic beta cells through the expression of angiotensin-converting enzyme 2 (ACE2) receptors, impairing insulin production, and consequently, either worsening DM or developing new-onset DM [8]. Insulin resistance due to higher levels of interleukin-6 and tumor necrosis factor-alpha in patients with severe COVID-19 could be another probable explanation for developing DM [9].

Despite this, many recently published data entailed the effect of previously diagnosed DM on the clinical course and outcome of COVID-19 [5,8]. Only a few data are available regarding the new-onset of DM among COVID-19 patients, its different types, its clinical course, and its outcome after the recovery from COVID-19. The purpose of this work was to determine the frequency of newly diagnosed DM and its different types among COVID-19 patients, and to assess the infection outcome and glycemic control during the study.

## 2. Methods

### 2.1. Study Population and Recruitment

In this biphasic cross-sectional/prospective study, after excluding known cases of DM, we included 570 confirmed COVID-19 patients who were admitted to Zagazig University Hospital and Zagazig General Hospital, from 1 April 2020 to 31 May 2020. Exclusion criteria included age <18 years old, pregnancy, unconfirmed cases of COVID-19, and previously diagnosed cases of DM.

### 2.2. Patient Assessment

All patients underwent thorough clinical and laboratory assessment and chest computerized tomography (CT). The following laboratory measures were recorded in all participants on admission: complete blood count measured using Sysmex XN-2000 autoanalyzer (Siemens Diagnostic, Erlangen, Germany) and erythrocyte sedimentation rate (ESR) measured using Vision B analyzer (YHLO Biotech diagnostic, Shenzhen, China). Biochemical blood tests included FPG, HbA1c, C-reactive protein (CRP), serum total bilirubin, albumin, Transaminases (ALT, AST), LDH, creatinine, and urea nitrogen measured using dedicated reagent on Cobas c702/8000 (Roche diagnostic, Mannheim, Germany), and D-dimer measured on Cobas c501/6000 (Roche diagnostic, Germany). Serum ferritin, serum fasting insulin, and C-peptide were measured on Cobas c602/8000 (Roche diagnostic, Germany) in newly diagnosed diabetic subjects. COVID-19 diagnosis was confirmed by reverse transcription-polymerase chain reaction (RT-PCR) using nasal and pharyngeal swabs.

### 2.3. Study Design and Setting

According to Chinese National Health Committee diagnostic guidelines for COVID-19, disease severity was graded as either mild/moderate (minimal symptoms and negative chest CT findings) or severe (extensive clinical manifestations and positive CT findings) [10]. According to the American Diabetes Association, newly diagnosed DM was defined as either new-onset DM (no preceding history of DM with fasting plasma glucose [FPG] ≥ 126 mg/dL or random blood glucose [RBG] ≥ 200 mg/dL and HbA1c < 6.5%) or previously undiagnosed DM (FPG ≥ 126 mg/dL or RBG ≥ 200 mg/dL and HbA1c ≥ 6.5% or HbA1c ≥ 6.5% only [11]. In the first (cross-sectional) phase of the study, based on mean readings of FPG on the first day of hospital admission, all participants were classified into two main groups: group I (non-diabetic, FPG < 126 mg/dL) or group II (newly discovered diabetic, FPG ≥ 126 mg/dL). According to measurements of HbA1c and serum fasting insulin and C-peptide on admission, group II patients were further classified into group IIA (newly discovered pre-existing DM; HbA1c ≥ 6.5), group IIB (new-onset type 1 DM; HbA1c < 6.5, low fasting insulin and low C-peptide), or group IIC (new-onset type 2 DM; HbA1c < 6.5, normal fasting insulin and normal C-peptide). The outcome of COVID-19 infection was recorded either as recovery or mortality in different groups. In the second (prospective) phase of the study, patients with new-onset DM (groups II B,C) were followed for three months (from diagnosis of DM), even after discharge from the hospital, with weekly based outpatient visits, repeated testing of FPG, and recording of anti-diabetic treatment.

### 2.4. Statistical Analysis

Statistical calculations were conducted using SPSS version 21.0 for Windows (SPSS Inc., Chicago, IL, USA). If the continuous variables were parametric, they were expressed as mean ± SD, meanwhile if they were nonparametric, the median was the method of expression. In addition, categorical variables were expressed as numbers and percentages. Student’s t-test, Mann–Whitney U test, Fisher’s exact test, Pearson’s Chi-squared test, and logistic regression model were among the appropriate tests utilized. Statistical significance was considered at *p* < 0.05.

## 3. Results

### 3.1. Cross-Sectional Phase

This study comprised 570 confirmed COVID-19 patients. The mean age of the study population was 47.9 ± 10.9 years, and 317 patients (55.5%) were males. Diabetes was newly defined in 77 (13.5%) patients. Our results showed that there were significant differences between newly diagnosed diabetic patients and non-diabetic patients regarding body mass index (BMI) and family history of DM (*p* < 0.001, for both). Fasting blood glucose (208.3 ± 109.9) and glycated hemoglobin (5.7 ± 0.8) were found to be significantly higher in the newly diagnosed diabetic patients (*p* < 0.001). In terms of onset symptoms in the newly diagnosed diabetic patients, 70 (90.9%) patients exhibited symptoms of fever; the other common symptoms were cough in 70 (90.9%), dyspnea in 66 (85.7%), and diarrhea in 11 (14.3%) patients. There were several differences in laboratory findings between newly diagnosed diabetic patients and non-diabetic patients, including higher levels of C-reactive protein (CRP), lactate dehydrogenase (LDH), ferritin, and D-dimer in newly diagnosed DM (*p* < 0.001, for all). The study enrolled 297 severe COVID-19 cases (52.1%) and 273 mild/moderate cases (47.9%). Out of all cases of newly diagnosed DM, 89.6% had a severe infection (69/77), which was significantly higher than that among non-diabetic patients (*p* < 0.001). A total number of 62 patients died during the study (10.9%) from COVID-19 sequelae. Mortality was significantly higher among diabetic subjects (18.2%) than non-diabetic subjects (9.7%) (*p* < 0.001) (Table 1).

**Figure 1:**
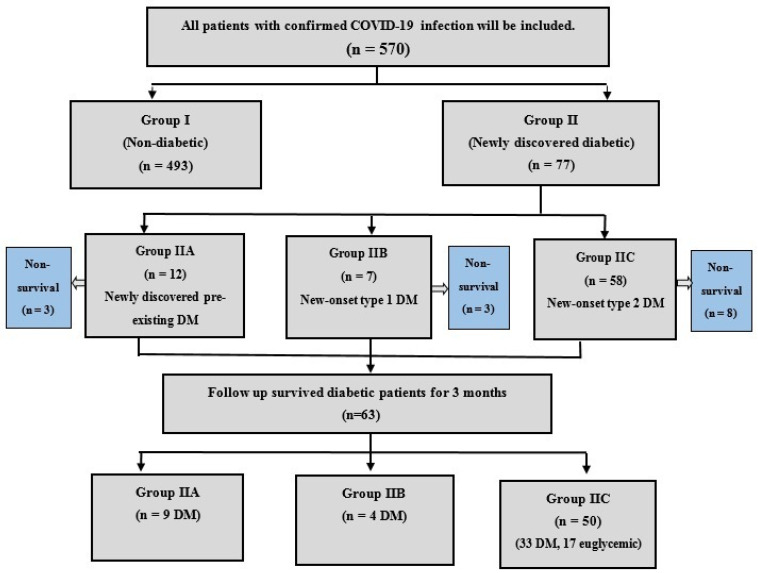
Flow diagram of study participants.

Out of 77 diabetic patients, newly discovered pre-existing DM was defined in 12 (2.1%) patients, new-onset type 1 DM developed in seven (1.2%) patients, and 58 (10.2%) patients had new-onset type 2 DM (Figure 1).

As displayed in Table 2, HbA1C level was significantly elevated in group IIA (7.2 ± 0.4), compared with group IIB (5.3 ± 0.5) and group IIC (5.4 ± 0.5) (*p* < 0.001). However, fasting insulin and C-peptide levels were much lower in group IIB (3.6 ± 1.3, 0.3 ± 0.1) compared with group IIA (33.4 ± 9.2, 3.5 ± 1) and group IIC (38.1 ± 9.1, 3.6 ± 0.8) (*p* < 0.001). There were significant differences among groups IIA, IIB, and IIC regarding age, BMI, FPG, HbA1c, fasting insulin, and C-peptide (*p* < 0.001, for all). Four cases (57.1%) in group IIB showed positive urinary acetone with DKA on presentation. There were several differences in laboratory findings between groups IIA, IIB, and IIC, including higher levels of CRP, serum ferritin, and D-dimer in group IIB.

Serum CRP, ferritin, LDH, and D-dimer had significant positive correlations with newly diagnosed DM. In addition, age, BMI, severe COVID-19, and positive chest CT findings had significant positive correlations with newly diagnosed DM (*p* < 0.001, for all) (Table 3).

Table 4 shows the independent significant predictors of the presence of newly diagnosed DM among COVID-19 patients as determined by logistic regression analyses. Older age, higher BMI, elevated CRP, and elevated ferritin were the significant predictors (*p* < 0.001, for each).

Predictors of mortality among COVID-19 patients (62/570, 10.9%) comprised older age, hypertension, ischemic heart disease (IHD), development of DM, DKA on presentation, severe COVID-19 infection, positive Chest CT findings, elevated CRP, elevated ferritin, lymphopenia, and elevated D-dimer (*p* < 0.001, for each) (Table 5).

### 3.2. Follow-Up Phase (for DM)

All patients with newly diagnosed DM (*n* = 77) were given the appropriate anti-diabetic treatment (starting with insulin therapy). Four cases of DKA were diagnosed (all among type I DM patients) and were managed successfully in ICU. Out of 77 cases of newly diagnosed DM, 14 patients died shortly (1–19 days after diagnosis) from COVID-19 sequelae. Survived diabetic patients (*n* = 63) were followed for three months with repeated testing of FBS.

### 3.3. Study Endpoint (Glycemic Control after 3 Months)

Hyperglycemia and the need for anti-diabetic treatment (insulin therapy or oral drugs) persisted in 73% of surviving diabetic patients (46/63), whereas anti-diabetic treatment could be stopped in 17 patients (27%). Hyperglycemia persisted in all survived patients with pre-existing DM (*n* = 9). In survived subjects with new-onset DM types I and II (*n* = 54), hyperglycemia persisted in 37 patients (68.5%), including all four patients with DM type I and 66% of patients with DM type II (33/50).

## 4. Discussion

The COVID-19 outbreak is increasing rapidly throughout the world. The new virus responsible for this epidemic was named as SARS-CoV-2, which has now turned into a global catastrophe [12]. COVID-19 causes a novel pathophysiological alteration in glucose homeostasis (a combination of severe insulin resistance and insulin insufficiency), making COVID-19-related diabetes management difficult [13].

The purpose of this cross-sectional study was to determine the frequency of newly diagnosed DM and its different types among COVID-19 patients and to explore the infection outcome and glycemic control of newly diagnosed diabetic patients during the study.

In the first phase of this biphasic cross-sectional study, newly diagnosed diabetes (FPG > 126) was defined in 77 patients (13.5%). This agreed with several studies. Wang et al. reported that 29.1% (176/605) of COVID-19 patients with no previous diagnosis of DM had FPG ≥ 126 mg/dL [14]. Smith et al. reported that COVID-19 is associated with increased FPG and 15.8% of patients developed new-onset DM [15]. Guan et al. stated that DM was discovered in 7.4% of a cohort of COVID-19 hospitalized patients and appeared to be a risk factor for the severity of illness [16]. Finally, a meta-analysis of eight studies with more than 3700 patients showed a pooled proportion of 14.4% for newly diagnosed diabetes in COVID-19 hospitalized patients [17].

HbA1c was performed for all newly diagnosed diabetic patients to differentiate between new-onset and pre-existing DM; fasting insulin and C-peptide were also performed to differentiate between new-onset type 1 DM and new-onset type 2 DM. Accordingly, out of 77 newly diagnosed diabetic patients, newly discovered pre-existing DM was defined in 12 patients (2.1%), new-onset type 1 DM developed in 7 patients (1.2%), and 58 patients (10.2%) had new-onset type 2 DM. However, in some studies, HbA1c was not performed for all participants, so it was not possible to differentiate between new-onset and previously undiagnosed diabetes [14,18].

In the present study, as compared to non-diabetic patients, the newly diagnosed diabetic patients had significantly older age (57.7 ± 11.4, vs. 46.4 ± 10, *p* < 0.001), higher BMI (32 ± 9 vs. 25 ± 4.5, *p* < 0.001), and positive family history of diabetes (44.2% vs. 4.5%, *p* < 0.001). This was in agreement with Li H et al., who reported that COVID-19 patients with newly diagnosed DM and hyperglycemia were slightly older and obese [19].

In the current work, four patients (all with new-onset type 1 DM) presented with diabetic ketoacidosis (DKA) on admission. This was in agreement with Reddy et al., who stated that COVID-19 may accelerate DKA in those with new-onset or pre-existing DM [20].

Our study also revealed that patients with newly diagnosed DM had more severe infection symptoms such as fever, dyspnea, and cough, as well as elevated levels of inflammatory markers such as CRP, LDH, and ferritin than non-diabetic patients. Our results were in concordance with Li H et al., who stated that patients with newly diagnosed diabetes and hyperglycemia often had more severe symptoms as well as higher levels of inflammatory markers [19]. Infection with COVID-19 decreases ACE2 expression, resulting in hyperinflammation, cellular damage, and respiratory failure [21].

The results of the current study revealed that positive chest CT findings (89% vs. 46%, *p* < 0.001) and D-dimer (1.5 ± 2 vs. 0.9 ± 1.2, *p* < 0.001) were higher in diabetic group as compared to non-diabetic group.

Mortality within the COVID-19 patients of our study was significantly higher among newly diagnosed diabetic than non-diabetic patients (18.2% vs. 9.7%, *p* = 0.046). Likewise, one study from the United States found that COVID-19 patients who are diabetics had a markedly higher mortality than patients without diabetes (28.8% vs. 6.2%) [22]. Furthermore, Li H et al. reported that in patients with COVID-19, newly diagnosed DM is connected to increased mortality when compared to known DM and normal glucose levels [19]. Chronic hyperglycemia was linked to decreased immunity, and hyperglycemia was found to be an independent predictor of lower respiratory tract infection and poor prognosis [4].

Our logistic regression analysis showed that older age, higher BMI, elevated CRP, and elevated ferritin were the significant predictors of newly diagnosed DM among COVID-19 patients (*p* < 0.001).

The results of the current study revealed that increasing age, hypertension, IHD, and high levels of D-dimer were reported as important predictors of mortality among COVID-19 patients. This agreed with Zhou et al., who confirmed that the death rate was higher in elderly patients with COVID-19 [23]. Rodelo et al. stated that increased levels of D-dimer have been linked to 28-day mortality in patients with severe infection or sepsis in intensive care units [24]. A pooled analysis stated that hypertension is associated with a 2.5-fold increased risk of both severity and mortality in COVID-19 patients [25]. Moreover, Bonow et al. found that patients with underlying cardiovascular disease are more likely to have serious COVID-19 outcomes, including death, as we discovered in our study [26].

Out of the 77 patients with DM, 14 (18.2%) patients died shortly (1–19 days) due to COVID-19 sequelae. The remaining 63 patients were followed for three months. Hyperglycemia and the need for anti-diabetic treatment persisted in 46 (73%) patients, while 17 (27%) patients became euglycemic and did not need anti-diabetic treatment after recovery from the acute illness, indicating that they had stress-induced hyperglycemia, an adaptive immune-neurohormonal response to physiological stress.

To the best of our knowledge, this is the first study to assess the different types of newly diagnosed DM among COVID-19 patients and to explore the persistence of hyperglycemia over the study duration, as assessed by laboratory measurements and follow-up. As a contribution of this study, it raises the hypothesis for the development of a large-scale multinational study to assess the development and prevention of DM in COVID-19 patients. The relatively short follow-up duration was a limitation to this study.

## 5. Conclusions

A significant proportion of COVID-19 patients (13.5%) experienced the appearance of new-onset DM and expression of pre-existing DM during disease course, both being frequent with more severe infection. The newly diagnosed DM persisted for three months in about two-thirds of affected subjects. COVID-19 patients with newly diagnosed diabetes had high risk of mortality compared with COVID-19 patients without diabetes. Increasing age, hypertension, IHD, and high levels of D-dimer were reported as important predictors of mortality among COVID-19 patients. We recommend that a patient with COVID-19 infection have their blood glucose levels constantly checked for the emergence of full-blown diabetes. Newly diagnosed diabetes should be handled early and effectively.

## Figures and Tables

**Table 1:** Comparison between diabetic and non-diabetic groups regarding clinical, laboratory, and radiological differences.

Variables	No. (%), Mean ± SD or Median.			*p*
	All Participants (*n* = 570)	Non-Diabetic Patients (Group I, *n* = 493)	Newly Diagnosed Diabetic Patients (Group II, *n* = 77)	
Age (years)	47.9 ± 10.9	46.4 ± 10	57.7 ± 11.4	<0.001
Male gender	317 (55.5%)	276 (56%)	41(53.2)	0.712 *
BMI	26 ± 5.9	25 ± 4.5	32 ± 9	<0.001 **
Hypertensive	45 (7.9%)	36 (7.3%)	9 (11.7%)	0.178 *
IHD	19 (3.3%)	12 (2.4%)	7 (9.1%)	0.008 *
Family history of DM	56 (9.8%)	22 (4.5%)	34 (44.2%)	<0.001 *
Severe COVID-19	297 (52.1%)	228 (46.2%)	69 (89.6%)	<0.001 *
Fever	327 (57.4%)	257 (52.1%)	70 (90.9%)	<0.001 *
Cough	312 (54.7%)	242 (49.1%)	70 (90.9%)	<0.001 *
Dyspnea	301 (52.8%)	235 (47.7%)	66 (85.7%)	<0.001 *
Diarrhea	78 (13.7%)	67 (13.6%)	11 (14.3%)	0.859 *
FPG (mg/dL)	105.9 ± 57.9	89.9 ± 10.3	208.3 ± 109.9	<0.001 **
HbA1C	5.4 ± 0.6	5.4 ± 0.6	5.7 ± 0.8	<0.001
Positive urinary acetone	4 (0.7%)	0	4 (5.2%)	<0.001 *
DKA on presentation	4 (0.7%)	0	4 (5.2%)	<0.001 *
Hemoglobin (g/dL)	11.8 ± 1.4	11.7 ± 1.4	11.9 ± 1.4	0.234
Platelets count (×10^3^/mm^3^)	191.3 ± 50.6	192.3 ± 50.1	184.9 ± 53.1	0.230
WBCs (×10^3^/mm^3^)	6.4 ± 2.5	6.4 ± 2.6	6.2 ± 2.3	0.511
Lymphocytes (×10^3^/mm^3^)	2.1 ± 1.1	2.2 ± 1.1	1.5 ± 0.8	<0.001 **
Absolute lymphopenia (<1 × 10^3^/mm^3^)	169 (29.6%)	123 (24.9%)	46 (59.7%)	<0.001 **
CRP (mg/dL)	38.8 ± 23.8	36.3 ± 19.9	55.4 ± 37.2	0.009 **
ESR (mm/h)	41.3 ± 14	41.1 ± 13.9	42.6 ± 14.5	0.389
Serum ferritin (ng/mL)	236 ± 170.3	217.9 ± 150.4	351.7 ± 234.6	<0.001 **
LDH (IU/L)	246.2 ± 80.5	239.9 ± 77.4	287 ± 88.7	<0.001 **
D-dimer (μg/mL)	1 ± 1.4	0.9 ± 1.2	1.5 ± 2	<0.001 **
Serum creatinine (mg/dL)	1 ± 0.3	1 ± 0.3	1 ± 0.2	0.727
Blood urea (mg/dL)	36.6 ± 25.5	36.5 ± 25.3	37 ± 26.3	0.893
INR	1.1 ± 0.2	1.1 ± 0.2	1.1 ± 0.1	0.098
Serum albumin (g/dL)	3.6 ± 0.5	3.6 ± 0.5	3.7 ± 0.4	0.352
Serum total bilirubin (g/dL)	1.1 ± 0.2	1.1 ± 0.2	1.1 ± 0.1	0.058 **
ALT (IU/L)	33.9 ± 23.7	33.4 ± 23.2	36.6 ± 26.7	0.278
AST (IU/L)	56.2 ± 37.7	55.4 ± 36.8	60.8 ± 43	0.249
Positive chest CT findings	297 (52.1%)	228 (46.2%)	69 (89.6%)	<0.001 *
Deceased	62 (10.9%)	48 (9.7%)	14 (18.2%)	0.046 *

Unless otherwise indicated, data represent the mean ± SD with the range in parenthesis. No: number; SD: standard deviation; BMI: body mass index; IHD: ischemic heart disease; WBCs: white blood cells; CRP: C-reactive protein; ESR: erythrocyte sedimentation rate; LDH: lactate dehydrogenase; IU: international unit; INR: international normalized ratio; ALT: alanine aminotransferase; AST: aspartate aminotransferase; CT: computerized tomography. *: Fisher’s Exact Test; **: Mann–Whitney Test.

**Table 2:** Clinical, laboratory, and radiological differences among different diabetic subgroups (*n* = 77).

Variables	No. (%), Mean ± SD or Median.			*p*
	Pre-Existing DM (Group IIA, *n* = 12)	New-Onset Type 1 DM (Group IIB, *n* = 7)	New-Onset Type 2 DM (Group IIC, *n* = 58)	
**Age (years)**	58.3 ± 8.7	36 ± 8.6	60.1 ± 9.3	<0.001
Male patients	7 (58.3%)	4 (57.1%)	30 (51.7%)	0.895
BMI	29.1 ± 6.7	21.4 ± 2.4	33.9 ± 9	<0.001 **
Hypertensive	1 (8.3%)	0	8 (13.8%)	0.520
IHD	0	0	7 (12.1%)	0.283
Family history of DM	5 (41.7%)	2 (28.6%)	27 (46.6%)	0.652
Severe COVID-19	8 (66.7%)	7 (100%)	54 (93.1%)	0.015
FPG (mg/dL)	194.3 ± 64.3	473.4 ± 124.4	179.2 ± 64.5	<0.001 **
HbA1C	7.2 ± 0.4	5.3 ± 0.5	5.4 ± 0.5	<0.001
Fasting insulin (mIU/L)	33.4 ± 9.2	3.6 ± 1.3	38.1 ± 9.1	<0.001
C-peptide (ng/mL)	3.5 ± 1	0.3 ± 0.1	3.6 ± 0.8	<0.001 **
Positive urinary acetone	0	4 (57.1%)	0	<0.001
DKA on presentation	0	4 (57.1%)	0	<0.001
Hemoglobin (g/dL)	12.2 ± 1.9	10.9 ± 1.4	12 ± 1.3	0.119
Platelets count (×10^3^/mm^3^)	154.9 ± 32.4	190.1 ± 88.2	190.4 ± 50.1	0.103
WBCs (×10^3^/mm^3^)	6.3 ± 2.5	6.9 ± 2.9	6.2 ± 2.3	0.747
Lymphocytes (mean ± SD, ×10^3^/mm^3^)	1.7 ± 0.9	1 ± 0.3	1.5 ± 0.8	0.282
Absolute lymphopenia (<1 ×10^3^/mm^3^)	6 (50%)	6 (85.7%)	34 (58.6%)	0.291
CRP (mg/L)	55.7 ± 31	113.1 ± 56.9	48.4 ± 29.2	0.004 **
ESR ((mm/h)	39.5 ± 15.3	52.7 ± 14.4	42 ± 14.1	0.130
Serum ferritin (ng/mL)	318.9 ± 177.9	832 ± 252.7	300.6 ± 171.2	0.002 **
LDH (IU/L)	256.1 ± 82.5	360 ± 100.8	284.6 ± 85.1	0.100 **
D-dimer (μg/mL)	0.9 ± 0.5	5 ± 5.3	1.2 ± 0.9	0.007 s**
Serum creatinine (mg/dL)	1 ± 1.3	1.1 ± 0.3	1 ± 0.2	0.336
Blood urea (mg/dL)	29 ± 19	49.3 ± 37.6	37.1 ± 26	0.272
Positive chest CT findings	8 (66.7%)	7 (100%)	54 (93.1%)	0.015
Deceased	3 (25%)	3 (42.9%)	8 (13.8%)	0.136

Unless otherwise indicated, data represent the mean ± SD with the range in parenthesis. No: number; SD: standard deviation; BMI: body mass index; IHD: ischemic heart disease; WBCs: white blood cells; CRP: C-reactive protein; ESR: erythrocyte sedimentation rate; LDH: lactate dehydrogenase; IU: international unit; CT: computerized tomography. **: Kruskal–Wallis Test.

**Table 3:** Correlation between presence of newly diagnosed DM and different clinical and laboratory parameters (*n* = 570).

Variables	R	*p*
Age (years)	0.354	<0.001 *
BMI	0.312	<0.001 **
Severe COVID-19	0.297	<0.001 **
Lymphocytes (×10^3^/mm^3^)	−0.236	<0.001 *
Absolute lymphopenia (<1 × 10^3^/mm^3^)	0.260	<0.001 *
CRP (mg/L)	0.186	<0.001 **
Serum ferritin (ng/mL)	0.222	<0.001 **
LDH	0.191	<0.001**
D-dimer (μg/mL)	0.202	<0.001 **
Positive chest CT findings	0.297	<0.001 **

r: correlation coefficient; BMI: body mass index; CRP: C-reactive protein; LDH: lactate dehydrogenase; IU: international unit; CT: computerized tomography. *: Pearson’s correlation; **: Spearman’s correlation.

**Table 4:** Logistic regression analysis for predictors of presence of newly diagnosed DM among COVID-19 patients (*n* = 570).

Variables	B	Exp (B)	95% C.I. for Exp (B)		*p*
			Lower	Upper	
COVID-19 severity	−1.300	0.272	0.096	0.776	0.015
Age	0.081	1.084	1.047	1.123	<0.001
BMI	0.175	1.192	1.123	1.265	<0.001
Lymphocytes	−0.562	0.570	0.329	0.988	0.045
Lymphopenia	0.975	2.650	0.859	8.176	0.090
CRP	0.023	1.024	1.010	1.037	<0.001
Ferritin	0.003	1.003	1.001	1.004	<0.001
LDH	−0.002	0.998	0.994	1.003	0.456
D. dimer	0.020	1.020	0.824	1.264	0.854

CI: confidence interval; BMI: body mass index; CRP: C-reactive protein; LDH: lactate dehydrogenase.

**Table 5:** Predictors of mortality among all participants (62/570, 10.9%).

Variables	No. (%), Mean ± SD or Median.		*p*
	Survived Patients (*n* = 508)	Died Patients (*n* = 62)	
Age (years)	47.4 ± 10.9	52.2 ± 9.6	<0.001
Male gender	279 (54.9%)	38 (61.3)	0.341
BMI	25.9 ± 5.6	26.7 ± 7.8	0.284
Hypertensive	33 (6.5%)	12 (19.4%)	<0.001 *
IHD	14 (2.8%)	5 (8.1%)	0.028 *
Newly discovered DM	63 (12.4%)	14 (22.6%)	0.027
Type of DM			
Pre-existing DM	9 (1.8%)	3 (4.8%)	0.012 *
DM type 1	4 (0.8%)	3 (4.8%)	
DM type 2	50 (9.8%)	8 (12.9%)	
Severe COVID-19	247 (48.6%)	50 (80.6%)	<0.001
FPG (mg/dL)	103.5 ± 49.9	125.5 ± 100.5	0.179 **
HbA1C	5.4 ± 0.6	5.4 ± 0.6	0.966
DKA on presentation	2 (0.4%)	2 (3.2%)	<0.012 *
Hemoglobin (g/dL)	11.8 ± 1.4	11.7 ± 1.1	0.455
Platelets count (×10^3^/mm^3^)	192.1 ± 50.5	185.1 ± 51.2	0.305
WBCs (×10^3^/mm^3^)	6.5 ± 2.6	5.9 ± 2.4	0.113
Lymphocytes (×10^3^/mm^3^)	2.3 ± 1.1	1.2 ± 0.9	<0.001 **
Absolute lymphopenia (<1 × 10^3^/mm^3^)	116 (22.8%)	53 (85.5%)	<0.001 *
CRP (mg/dL)	36.2 ± 20.2	60.9 ± 36.9	<0.001 **
ESR (mm/h)	41.4 ± 14.1	40.0 ± 13.4	0.458
Serum ferritin (ng/mL)	223.4 ± 157.2	338.7 ± 230.5	<0.001 **
LDH (IU/L)	237.7 ± 75.5	316.2 ± 86.6	<0.001 **
D-dimer (μg/mL)	0.9 ± 0.9	1.9 ± 3.1	<0.001 **
Serum creatinine (mg/dL)	1 ± 0.3	1 ± 0.3	0.610
Blood urea (mg/dL)	36.1 ± 24.8	40.3 ± 30.5	0.367 **
INR	1.1 ± 0.2	1.2 ± 0.2	0.321
Serum albumin (g/dL)	3.6 ± 0.5	3.6 ± 0.5	0.328
Serum total bilirubin (g/dL)	1.1 ± 0.2	1.1 ± 0.2	0.764 **
ALT (IU/L)	34.4 ± 24.1	29.6 ± 19.5	0.269 **
AST (IU/L)	56.9 ± 38.7	50.2 ± 27.7	0.435 **
Positive chest CT findings	247 (48.6%)	50 (80.6%)	<0.001

Unless otherwise indicated, data represent the mean ± SD with the range in parenthesis. No: number; SD: standard deviation; BMI: body mass index; IHD: ischemic heart disease; WBCs: white blood cells; CRP: C-reactive protein; ESR: erythrocyte sedimentation rate; LDH: lactate dehydrogenase; IU: international unit; INR: international normalized ratio; ALT: alanine aminotransferase; AST: aspartate aminotransferase; CT: computerized tomography. *: Fisher’s Exact Test; **: Mann–Whitney Test.

## Data Availability

Not applicable.
